# Patterns of Recurrent Disease in Cervical Cancer

**DOI:** 10.3390/jpm12050755

**Published:** 2022-05-06

**Authors:** Maura Miccò, Michela Lupinelli, Matteo Mangialardi, Benedetta Gui, Riccardo Manfredi

**Affiliations:** 1 Dipartimento Diagnostica per Immagini, Radioterapia Oncologica ed Ematologia, Fondazione Policlinico Universitario A. Gemelli IRCCS, 00168 Rome, Italy; maura.micco@policlinicogemelli.it (M.M.); riccardo.manfredi@policlinicogemelli.it (R.M.); 2 Dipartimento Universitario di Scienze Radiologiche ed Ematologiche, Università Cattolica del Sacro Cuore, 00168 Rome, Italy; michela.lupinelli01@icatt.it (M.L.); matteo.mangialardi01@icatt.it (M.M.)

**Keywords:** cervical cancer, recurrence, MRI, personalized approach, CT

## Abstract

Uterine cervical cancer is one of the most common causes of cancer-related deaths among women worldwide. Patients with cervical cancer are at a high risk of pelvic recurrence or distant metastases within the first few years after primary treatment. However, no definitive agreement exists on the best post-treatment surveillance in these patients. Imaging may represent an accurate method of detecting relapse early, right when salvage treatment could be effective. In patients with recurrent cervical cancer, the correct interpretation of imaging may support the surgeon in the proper selection of patients prior to surgery to assess the feasibility of radical surgical procedure, or may help the clinician plan the most adaptive curative therapy. MRI can accurately define the extension of local recurrence and adjacent organ invasion; CT and 18F-FDG PET/CT may depict extra-pelvic distant metastases. This review illustrates different patterns of recurrent cervical cancer and how imaging, especially MRI, accurately contributes towards the diagnosis of local recurrence and the assessment of the extent of disease in patients with previous cervical cancer. Normal post-therapy pelvic appearance and possible pitfalls related to tissue changes for prior treatments will be also illustrated.

## 1. Introduction

Despite significant advances in screening, detection, and treatment of cervical lesions, cervical carcinoma (CC) is still a growing global burden and a significant public health problem. Primary treatments for CC have a cure rate of approximately 95% in early-stage disease and 40–60% in locally advanced disease [1]. Recurrent CC is defined as local tumor re-growth or development of lymph nodal or distant metastases at least six months after the primary lesion has regressed [2]. The most frequent sites of recurrence can be classified as follows [3]:

1. *Local or central-pelvic* (including recurrence in the vaginal vault alone, cervix, uterus, parametria);
2. *Regional* (with or without vaginal involvement) defined as *anterior*, invading bladder, ureters, urethra, or as *posterior*, invading rectum, anal sphincter, or as *lateral*, invading pelvic side wall, vessels and nerves, or involving pelvic lymph nodes stations;
3. *Distant*, including infra-diaphragmatic (para-aortic lymph nodes) or supra-diaphragmatic nodal recurrence, or distant organ metastasis (lungs, liver) [4].

Management of recurrent CC depends on previous therapeutic approaches, on the site, and on the extent of recurrence, and should be discussed in a multidisciplinary team. Recurrence following primary *surgery* has been reported to be about 27% [5]. Rates following fertility-sparing radical trachelectomy are favorable, with a figure of 4% [5]. Patients who were previously treated with concomitant *chemoradiotherapy* (CRT) demonstrated a recurrence rate of 32% [6]. An Italian multicenter retrospective study, including 327 women with recurrent CC, showed that the most frequent sites of recurrence are local (on vaginal vault) and regional [7]. In the assessment of 564 patients who underwent radical hysterectomies for CC, Webb et al. noted relapses in 104 patients, and the most frequent recurrence site after radical surgery was the central-pelvic type [8].

Distant failure was the predominant pattern of relapse seen in patients with locally advanced CC undergoing CRT, as revealed in the Kobayashi and in the Sasidharan series [9,10]. In 2019, the Retro–EMBRACE study evaluated 731 patients from 12 institutions who were treated with definitive external beam radiation therapy (EBRT) and concomitant CT, followed by image-guided adaptive brachytherapy (IGABT). The study revealed how implementation of IGABT has improved pelvic control and unmasked occult distant disease, making distant-alone relapse the predominant form of failure [11].

Careful patient selection and a comprehensive assessment of recurrent disease are of paramount importance to tailor salvage treatment. Imaging may be crucial in the patients selection process. Magnetic resonance imaging (MRI) has shown to be reliable in differentiating recurrence from post-treatment changes in pelvises previously treated for gynecologic tumors. Furthermore, MRI is accurate in the evaluation of recurrent tumor extent and organ invasion. Computed tomography (CT) and 18F-fluorodeoxyglucose (FDG) positron emission tomography (PET)/CT scans have also been used for the evaluation of recurrent CC. In this study, we detail how imaging accurately contributes towards the diagnosis of local recurrence and the assessment of the extent of disease, showing specific imaging patterns of recurrent CC that are related to the type of previous treatment and tumor site, focusing on MRI and CT appearances. The viewer will learn how to interpret MRI and CT findings in recurrent CC to help clinicians to assess the feasibility of the most adaptive and personalized salvage treatments.

## 2. Imaging Surveillance

Typically, one-third of CC will recur within the first 2 years after initial treatment [4]. Moreover, in women with a history of treated CC, many recurrences may be asymptomatic [12]. The National Comprehensive Cancer Network (NCCN) guidelines recommend follow-up evaluation every 3–6 months for the first 2 years, followed by every 6 months for the next 3 years in patients treated for CC [13]. However, there are no formal recommendations in the reviewed literature for routine imaging surveillance for those patients. 

According to the Society of Gynecologic Oncology, imaging modalities may be indicated based on patient symptoms (abdominal and pelvic pain, leg pain or lymphedema, vaginal bleeding or discharge, urinary symptoms, cough and weight loss), on findings, on physical examination, or on increasing tumor markers (squamous cell carcinoma antigen, SCC or cancer antigen, Ca-125) [13,14]. Furthermore, the presence of symptoms may raise the suspicion of tumor recurrence and lead to unscheduled evaluation. Thus, counseling patients about signs and symptoms remains an important part of survivorship care [13].

The use of cytologic evaluation should be eliminated or limited to once a year, as it has shown low detection rates [13,15].

Elit et al. reviewed 17 retrospective studies to determine the optimal follow-up for patients with treated CC [15]. Asymptomatic recurrent disease was detected using physical examination in 29–71%, using chest X-rays in 20–47%, CT in 0–34% and vaginal vault cytology in 0–17% of patients, respectively [15]. In other studies, pelvic MRI, CT and 18F-FDG PET/TC scans were found to be effective in detecting recurrence in patients with clinical suspicion of recurrence [5,13,16]. 18F-FDG PET/CT is used to determine the evidence of distant disease and may provide predictive biomarkers of progression-free and overall survival [17]. Thorax–abdomen–pelvis contrast enhanced CT scan has little use in identifying local recurrence, but it plays an important role in the detection of distant metastases [2]. 

MRI is the best imaging technique for detecting local recurrence after treatment. The pooled sensitivity and specificity of MRI in CC recurrence were reported to be 82–100% and 78–100%, respectively [18]. Furthermore, pelvic MRI is usually performed in the follow-up of patients submitted to trachelectomy, who should be evaluated at 6 months and then annually for 2–3 years [19]. Pelvic MRI should be performed at least 6 months after the end of treatment, to differentiate between regular post-treatment changes and tumor re-growth. MRI should consist of at least two T2-weighted images (WI) in sagittal, axial oblique, or coronal oblique orientation (respectively along short and long axis of the remaining cervix), for detection of pelvic and para-aortic lymph nodes from renal veins to symphysis. Intravenous administration of gadolinium chelates is recommended for follow-up, and diffusion-weighted sequences (DWI) with a b-value between 0 and 1000 s/mm2 are useful to evaluate residual lesion and regional metastasis [20]. The basic imaging protocol for patients with suspected recurrence from gynecological malignancies is reported in Table 1. 

## 3. Normal Imaging Findings

To recognize pathological findings of recurrence and avoid false positive diagnosis, the MRI post-treatment appearance of the pelvis should be well known. 

**Figure 1:**
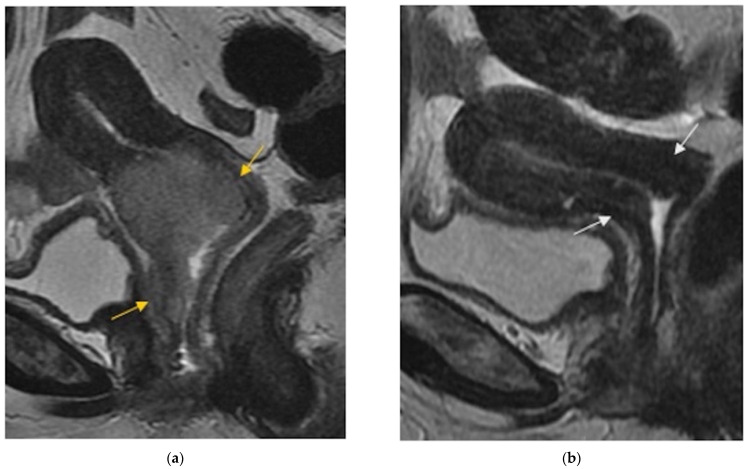
(**a**) Sagittal FSE T2-weighted image shows soft tissue with high signal intensity consistent with a cervical tumor extended to the vagina (arrows). (**b**) Sagittal FSE T2-weighted image shows reconstitution of the normal T2-weighted hypointense cervical stroma (arrows), with disappearance of the tumoral mass after chemoradiation therapy.

In patients who underwent laparoscopic vaginal radical trachelectomy—end-to-end anastomosis of uterine corpus to vaginal vault, especially the posterior neofornix—may assume a nodular configuration, and vaginal walls may be thicker in the first 6 months on T2WI [21]. In patients treated with radical hysterectomy, the normal vaginal cuff has a hypointense muscular wall on T2WI, with well-defined and regular margins [2]. Para-vaginal dissection may cause vaginal wall thickening due to transient edema that is more prominent in the first 6 months [2]. In the evaluation of patients submitted to CRT, satisfactory response to treatment is seen as a decrease in size and in signal intensity of tumor area on MR images, with reconstitution of the normal T2WI hypointense cervical stroma, which represents the most reliable indicator of complete response to therapy (Figure 1). 

However, in the first 6 months after CRT, acute radiation-induced edema and inflammation are possible findings, typically T2W hyperintense at MR images, making differential diagnosis with tumoral recurrence harder. The diagnostic performance of MR imaging may be improved by administration of contrast media and DWI sequences. At MR imaging, recurrent tumor has intermediate-to-high T2W signal intensity, showing early enhancement (45–90 s) and abnormal diffusion restriction on DWI sequences, while post-radiation changes demonstrate only mild delayed enhancement [22]. Soft tissue and parametria may undergo fibrotic changes, appearing hypointense on T2WI after radiotherapy (RT) [23,24]. This post-radiation imaging appearance may mimic parametrial invasion. Intravenous administration of contrast material and DWI images can also help distinguish between radiation-induced parametrial fibrosis and recurrent disease. Abnormal findings in different types of recurrence are synthetized in Table 2.

## 4. Pelvic Recurrence

### 4.1. Local

**Figure 2:**
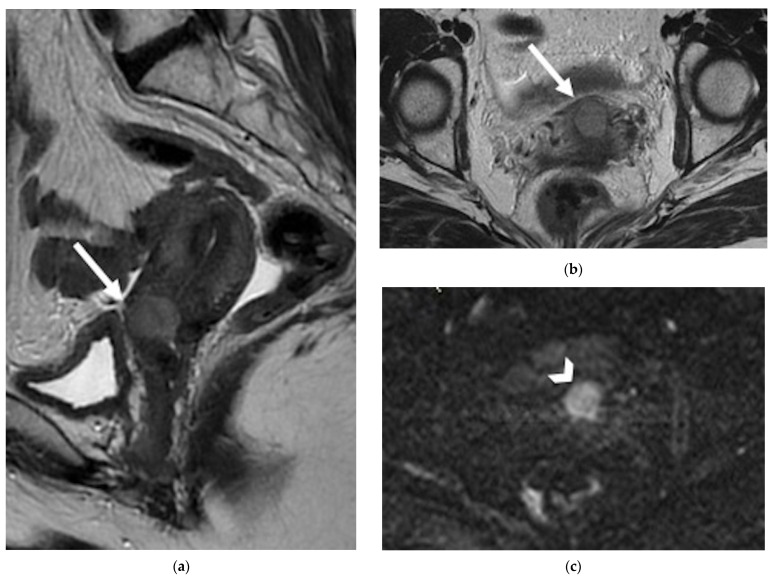
A 42-year-old patient with history of cervical cancer treated with chemoradiation therapy. (**a**) Sagittal and (**b**) axial FSE T2-weighted images show a nodular hyperintense lesion in the anterior aspect of the cervix, causing interruption of the hypointense cervical stroma, consistent with recurrence (arrows). (**c**) Axial diffusion-weighted image demonstrates restricted diffusion of the lesion (arrowhead).

**Figure 3:**
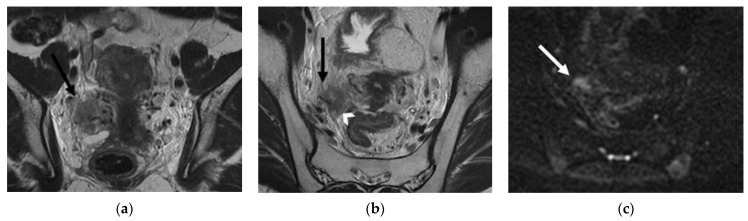
A 32-year-old woman who underwent previous chemoradiation therapy for cervical cancer. (**a**) Coronal and (**b**) axial FSE T2-weighted images show a soft-tissue mass in the right parametrium (black arrows). Note the contact with the right ureter embedded by the lesion (arrowhead). (**c**) Diffusion-weighted image shows focus of hyperintense signal into the lesion (white arrow). The finding was confirmed to be recurrence at surgery.

**Figure 4:**
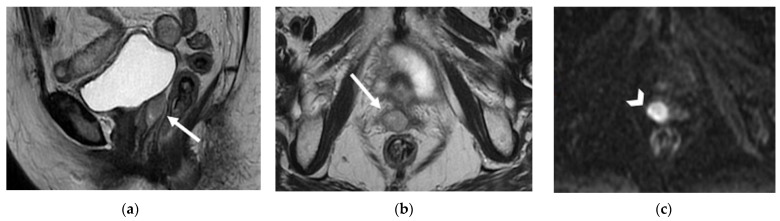
A 57-year-old patient who underwent radical hysterectomy with bilateral salpingo–oophorectomy for cervical cancer. (**a**) Sagittal and (**b**) axial FSE T2-weighted images show a nodular hyperintense lesion in the vaginal cuff (arrows), consistence with relapse. (**c**) Diffusion-weighted image demonstrates highly restricted diffusion of the lesion (arrowhead) in relation to hypercellularity.

About 30–45% of CC recurrences are central-pelvic type. Central recurrence may be located in the preserved cervix (Figure 2), parametria (Figure 3) or in the vaginal cuff in patients who have undergone prior hysterectomy (Figure 4).

On MRI, tumor recurrence may appear as a region of intermediate-to-high signal intensity on T2WI, on a background of established low-signal-intensity change reflecting radiation-induced fibrosis [23] (Figure 2). 

Relapse vaginal cuff lesions may appear as a mass-like tumor or as an infiltration along the vaginal wall, but they may also occur as skip lesions involving the distal vagina. On T2WI, tumor recurrence is seen as the loss of the linear, low-signal-intensity of the vaginal vault with an associated intermediate-to-high signal intensity soft-tissue mass [25] (Figure 4). Post-treatment fibrotic changes may alter the linear appearance of the vaginal vault, with adhesions between the vaginal vault and surrounding organs (i.e., rectum, bladder). The use of intravenous gadolinium contrast medium can help distinguish between recurrent disease versus post-treatment fibrosis, with maximum tumor enhancement occurring between 45 and 90 s after contrast administration [26]. On DWI, an active tumor appears as an area with a hyperintense signal on high-b-value images, associated with lower apparent diffusion coefficient (ADC) values on the ADC maps. Recurrent tumor may be recognized on contrast enhanced CT scans as a soft-tissue mass with early enhancement. However, MR imaging is superior to CT for delineating recurrent tumors because it provides superior soft-tissue contrast [27]. Recurrent pelvic CC appears as a focal area of increase FDG-uptake on 18F-FDG PET/CT images. 

### 4.2. Regional

#### 4.2.1. Anterior

**Figure 5:**
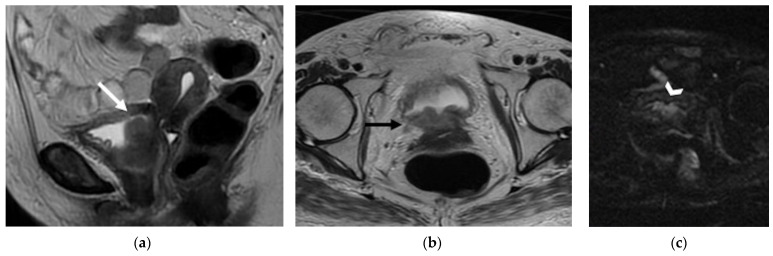
A 65-year-old woman who underwent chemoradiation therapy for cervical cancer. (**a**) Sagittal and axial (**b**) FSE T2-weighted image show an anterior pelvic recurrence, characterized by a soft-tissue mass infiltrating the posterior bladder wall extending into the bladder lumen (white and black arrow). (**c**) The lesion appears hyperintense on diffusion-weighted images (arrowhead).

In patients with pelvic recurrence, it is important to define whether the tumor is confined to the anterior pelvic compartment. Accurate detection of urinary bladder and urethral involvement has important implications for therapeutic management of patients. MR imaging has high positive and negative predictive values (up to 100%), with high accuracy (up to 95%) for assessment of bladder involvement. Anterior extension of pelvic recurrence may appear as a tumor infiltrating the bladder walls [28]. Associated ureteral obstruction can be due to central involvement of the bladder trigone at the ureteral orifice, resulting in hydronephrosis. High-resolution sagittal and axial oblique T2WI are ideal for assessment of bladder involvement (Figure 5). 

On T2WI, a clearly defined and uninterrupted fat plane between the tumor and the bladder is suggestive of the absence of organ involvement. Bladder involvement may be diagnosed when intermediate-to-high T2WI signal intensity soft-tissue mass interrupts the normal low T2WI signal intensity of the bladder wall (probable muscle invasion), or interrupts the normal high signal intensity of the mucosal layer on T2WI (mucosal involvement) [28]. On post-treatment surveillance, post-RT changes can be observed as a diffuse thickening of the bladder wall due to edema. On T2WI, bullous edema appears as a hyperintense band on the inner mucosal layer of the disrupted bladder wall, associated with incremented signal intensity of the bladder wall [29,30]. The use of intravenous gadolinium contrast medium and DWI may help differentiate bladder edema from true tumor infiltration. 

In case of pelvic recurrence invading the anterior compartment, the disease can extend below to the urethra. This structure is composed of tubular muscular layers, characterized by a low-signal-intensity concentric ringed appearance on T2WI. Urethral invasion is defined as a disruption of this bullseye aspect by a recurrence tumor [28]. 

Anterior recurrent lesions predispose the patient to the development of a fistulous tract between the vagina and inferior urinary tract. MRI is accurate in detecting and defining complex fistulas. The MRI appearance depends on whether it is filled with fluid, air, or a combination of the two. The use of multiplanar MR images allows a complete delineation of fistulas. At MRI, sagittal imaging planes are ideal for delineation of a vesicovaginal fistula, which typically appears as a linear fluid-filled tract on T2WI, associated with disruption of the posterior bladder wall and infiltrative soft-tissue-enhancing mass extended into the bladder lumen or in peri-vesical space in advanced recurrence disease [29,30]. The inclusion of fat-suppressed T2W acquisitions improves the detection of fluid-containing fistulas. On post-contrast images, fistulous tracks may be seen as linear peripheral enhancements connecting adjacent structures. 

Contrast enhanced CT and 18F-FDG PET/CT may help identify pelvic recurrent disease. The presence of a direct contact between recurrent tumor and adjacent organs, in the absence of a clearly defined fat plane on CT images, are diagnostic criteria for organ invasion. CT-urography and additional CT-cystography represent the preferred techniques to obtain a comprehensive evaluation of the urinary tract. On excretory-phase CT scans, contrast material may spread from the filling urinary bladder into the vaginal cuff or vagina. Maximum-intensity projection reconstructions may be beneficial to directly visualize the abnormal communication that leads to vaginal opacification.

#### 4.2.2. Posterior

**Figure 6:**
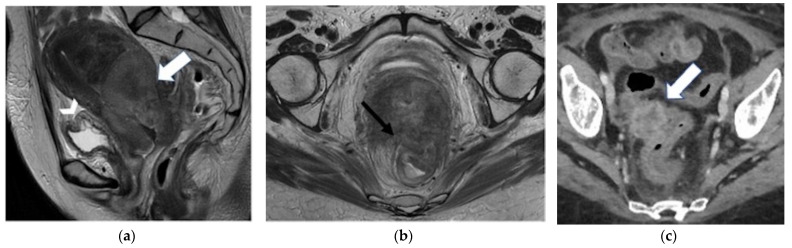
(**a**,**b**) A 46-year-old patient who underwent chemoradiation therapy for recent cervical cancer. (**a**) Sagittal and (**b**) axial FSE T2-weighted images show a soft-tissue mass in the cervix, extended posteriorly to the rectal wall (white arrow), causing luminal narrowing (black arrow). Note the bullous oedema of the posterior bladder wall mucosa (arrowhead). (**c**) A 58-year-old patient who underwent radical hysterectomy with BSO and chemoradiation therapy for cervical cancer. Axial post-contrast CT image shows a nodular mass in the right pelvis infiltrating the distal sigma (arrow).

Rectovaginal recurrent tumor has been reported in 17.3% of patients with previous CC [29]. Pelvic recurrence with posterior extension usually involves the rectum and it may be detected as an infiltrating, spiculated mass, causing rectal luminal narrowing (Figure 6a,b). More rarely, a tumor may involve the sigmoid colon (Figure 6c) or extend inferiorly to the anal sphincter. 

MRI is an accurate technique for evaluating rectum involvement, with a sensitivity of 71–100% and a specificity of 88–91% [22]. High-resolution small field-of-view sagittal and axial oblique T2WI are ideal for assessment of rectal involvement. On T2WI, rectal invasion is suspected in the case of interruption of the fatty plane between the recurrence and adjacent rectum (Figure 6). Tumor invading the rectal muscular wall and tumor nodules seen in the mucosal layer are an unequivocal sign of infiltration. The use of multiplanar MR images is crucial to better define the presence of visceral invasion. Post-contrast MR imaging may help distinguish between post-radiotherapy inflammation and disease recurrence [29]. A recto–vaginal fistula may develop either as a RT complication or in advanced recurrent disease. Rectovaginal fistulas may demonstrate interruptions of the vaginal and rectum muscularis with discontinuity of the intervening fat plane. In recurrent cervical cancer, rectum sigmoid junction is the common site of bowel invasion (Figure 6). MRI has been reported to be superior to CT in accurately predicting tumor invasion through the bowel wall [31]. Large bowel invasion may be identified on T2WI as a disruption of the three layers characteristic of the intestinal wall by an intermediate-to-hyperintense tumor, in the absence of a clear fat plane. Abnormal fistulous tracks containing air may be seen and are suggestive of diagnosis. Pelvic recurrence may also cause fixation of adjacent bowel loops, which can be associated with intestinal obstruction. In case of pelvic recurrence invading the posterior compartment, it is important to define whether the disease has reached and invaded the anal sphincter complex. The levator ani muscle (straight arrows) appears as a funnel-shaped muscular layer that extends from the obturator ani muscle to the anal canal. The most important components of the anal sphincter are the levator ani and the puborectal muscles. On coronal T2WI, it is essential to define whether the tumor is at or below the top border of the puborectal muscle. Less than 3 mm separation from the T2W hypointense muscles by the hyperintense tumor is a criterion for muscular invasion [32].

#### 4.2.3. Lateral

**Figure 7:**
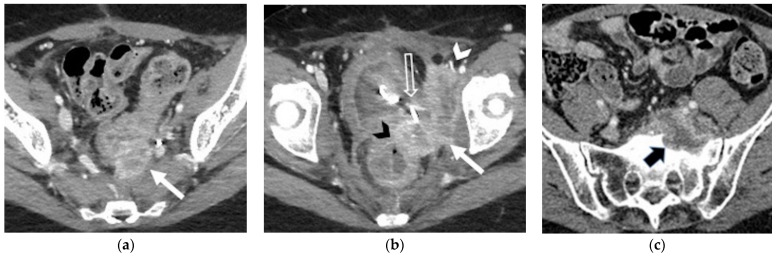
(**a**,**b**) A 36-year-old patient who underwent radical hysterectomy with bilateral salpingo–oophorectomy (BSO) and chemoradiation therapy for cervical cancer. Axial post-contrast CT images show pathological tissue infiltrating the left piriform muscle, the left internal obturator muscle (solid white arrow in (**a**,**b**)) and the external iliac vein (white arrowhead in (**b**)). Note the infiltration of the anterior rectal wall (black arrowhead in (**b**)), the bladder and the left ureteral orifice (with the ureteral stent) (open arrow in (**b**)). (**c**) A 58-year-old who underwent radical hysterectomy with BSO for cervical cancer. Axial post-contrast CT image shows pathological tissue infiltrating the left sacral promontory (black arrow).

In patients with extensive pelvic recurrence, it is important to determine whether the sidewall is involved by the tumor. Unilateral side wall invasion is more commonly seen than bilateral invasion. The MRI criteria for pelvic sidewall invasion are tumor extending within 3 mm and abutting the obturator internus or piriformis muscles with concomitant loss of fat planes. On post-contrast images, a tumor invading the pelvic side wall muscles usually shows heterogeneous enhancement. [28]. When these criteria are applied, MR imaging has a positive predictive value of up to 88% and negative predictive value of up to 97% for the presence of pelvic sidewall extension [33]. Furthermore, evidence of pelvic sidewall invasion on MRI has been shown to be associated with significantly shorter overall and recurrence free-survival [33]. CT images that show direct contact between tumor and pelvic muscles with no fat plane are indicative of pelvic sidewall invasion (Figure 7).

Other diagnostic imaging findings are: iliac vessels and ureters encased and narrowed by tumor (ureteral obstruction at the level of the tumor is considered to be an indicator of wall invasion); destruction of the pelvic cortical bone [29,30,34]. Imaging criteria used for determination of external iliac artery and vein vessel invasion are the presence of vessels surrounded or distorted by tumor, with a degree of tumor contact greater than 180°. Pelvic sidewall invasion may manifest with pain due to sciatic nerve infiltration [35]. Abnormal findings, which should raise suspicion of nerve invasion, include obliteration of fat planes around the nerves on T1WI, and hyperintense signal intensity at the nerve site on T2WI due to infiltrative edema [28]. Enhancement of peri-neural soft tissue on post-contrast fat-suppressed T1WI is considered a sign of nerve involvement. 

In case of pelvic side wall invasion, recurrent tumor may invade bony structures (Figure 7). In central-pelvic recurrence, ischio-pubic rami may be more frequently involved. In posterior recurrence, tumor growth may lead to infiltration of the pre-sacral space and sacral bone (Figure 7). On MRI, the diagnostic criteria for bone invasion are the loss of low signal-intensity cortex on both T1 and T2WI, and the replacement of the high signal intensity T1W marrow by the intermediate-to-high signal intensity tumor. Enhancing soft-tissue mass within the bone on gadolinium-enhanced fat-suppressed T1WI may also be identified. On CT, the pelvic bones may show lytic destructive changes due to the direct extension of the tumor [36]. 

## 5. Lymphadenopathies

There are three lymphatic pathways of drainage for the cervix. The cervical lymphatic network drains into the parametrial nodes; from there, the *lateral route* is to external iliac and obturator nodes, the *hypogastric route* is to the internal iliac nodes, and the *presacral route* is along the uterosacral ligament. All three routes lead to the common iliac nodes, from where tumors can involve the para-aortic nodes (PAN) [37]. PAN are common sites of distant relapse, described in 3–10.5% of patients after curative pelvic RT for CC [38].

The lymphatic spread of uterine cervical carcinoma can be divided into primary and secondary nodal groups. The first of these consists of the paracervical, parametrial, internal and external iliac, and obturator nodes, while the second includes the sacral, common iliac, inguinal, and PAN. When the secondary nodal group is involved, the prognosis worsens [39]. 

**Figure 8:**
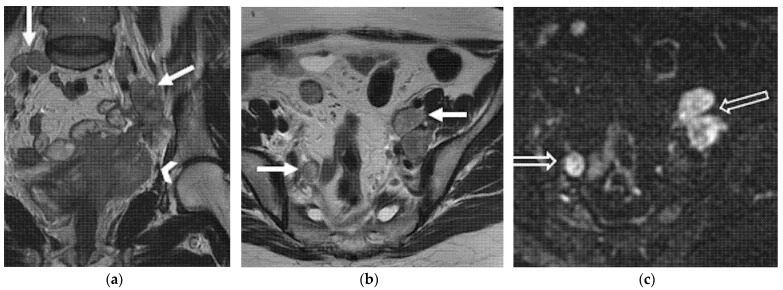
A 55-year-old patient who underwent radical hysterectomy with BSO and chemoradiation therapy for cervical cancer. (**a**) Coronal and (**b**) axial FSE T2-weighted images show multiple pelvic bilateral lymphadenopathies, with heterogenous signal intensity (arrows). Note the recurrent lesion in the vaginal cuff extended to the left parametrium (arrowhead in (**a**)). (**c**) The lymphadenopathies demonstrate restriction on diffusion-weighted image (open arrows).

MR and CT imaging determination of metastatic lymph nodes is based on size criteria, with a short-axis diameter of more than 1 cm, a round shape, irregular margins, and internal heterogeneity (Figure 8). 

18F-FDG PET/CT is better than the conventional imaging modalities in the detection of pathological lymph nodes in patients with gynecologic cancers, being able to detect metastatic normal-sized lymph nodes, with sensitivities of 75–100% and specificities of 87–100% [40,41]. A study by Walsh et al. revealed that in 38% of patients with central-pelvic recurrence, pelvic lymphadenopathy was also present [42]. Nodal metastases detected on imaging are considered a contraindication to radical surgery in patients with recurrent CC.

## 6. Distant Recurrence

### 6.1. Supra-Diaphragmatic Lymph Nodes

**Figure 9:**
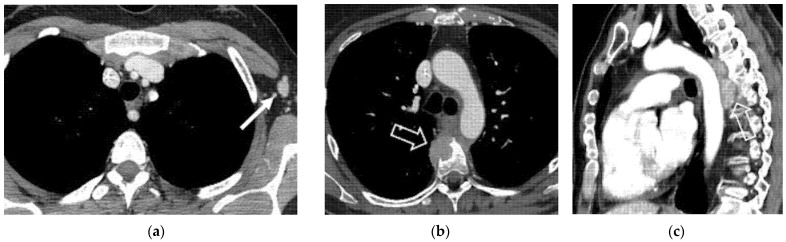
(**a**) A 40-year-old patient who underwent radical hysterectomy with BSO and chemoradiation therapy for cervical cancer. Axial post-contrast CT image shows left axillary lymph nodal relapse (solid arrow). (**b**,**c**) A 44-year-old patient who underwent radical hysterectomy with BSO and chemoradiation therapy for cervical cancer. (**b**) Axial and (**c**) sagittal post-contrast CT images show pathological mediastinal soft tissue infiltrating the vertebral soma (open arrows).

Supra-abdominal nodal recurrences, including para-bronchial, supra-clavicular, and axillary nodes, have been reported [29]. Mediastinal and hilar adenopathy and pleural metastases may be present in approximately one-third of patients with recurrent CC and lung metastasis [43]. CT is accurate when imaging modality in evaluation of presence metastatic extra-pelvic disease (Figure 9).

A sensitivity of 85.7–100% and specificity of 86.7–100% have been reported for 18F-FDG PET/CT in detection of recurrent disease outside the pelvis in patients with prior gynecologic cancer [44].

### 6.2. Abdominal and Extra-Abdominal Recurrence

Distant metastases from gynecologic cancer are usually due to recurrent disease. 

Abdominal recurrence occurs in the peritoneal cavity and solid organs, most frequently liver and adrenal gland. Liver metastases are present in one-third of patients with recurrent cervical disease [29]. Adrenal gland is commonly involved as well, more frequently in patients with adenocarcinoma rather than in patients with squamous cell CC. MR and CT imaging findings of hepatic and adrenal involvement are non-specific and indistinguishable from involvement by other primary malignancies. Spleen, pancreas, and kidneys are rarely involved [29,37]. Peritoneal carcinomatosis may be seen more frequently in patients with previous adenocarcinoma than in those with squamous cell CC, and appear as implants scalloping the liver contour, peritoneal nodularity, and soft-tissue masses with extrinsic compression of bowel loops. Ascites often occurs in association with peritoneal carcinomatosis. The lung is the most common site of distant disease; lung metastases usually manifest as multiple pulmonary nodules, but they may also appear as a solitary nodule or present cavitation; in this case, it is almost always associated with squamous cell histology. Lymphangitic carcinomatosis is seen in less than 5% of patients and appears as diffuse interstitial lung disease. Chest wall and endobronchial metastases are uncommon sites of relapse [43]. 

**Figure 10:**
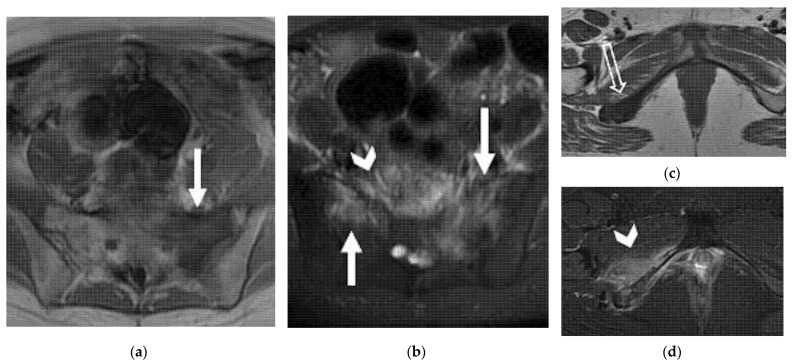
(**a**,**b**) A 56-year-old patient who underwent radical hysterectomy with BSO and chemoradiation therapy for cervical cancer. (**a**) Axial FSE T1 weighted and (**b**) axial FSE T2-weighted fat-sat images show osseous metastases in the sacrum, characterized by hypointense T1 and hyperintense T2 signal intensity (solid arrows). (**c**,**d**) A 41-year-old patient who underwent previous chemoradiation therapy for cervical cancer. (**c**) Axial FSE T1 weighted and (**d**) axial FSE T2-weighted fat-sat images show osseous metastases in the ischio–pubic right branch (open arrow in **c**). Note the oedema in the adjacent soft tissue and muscles (arrowheads in (**b**,**d**)).

The prevalence of osseous metastases in recurrent CC ranges from 15% to 29%. Metastases may appear on imaging as destruction of bone structure with an accompanying soft-tissue mass (Figure 10). 

**Figure 11:**
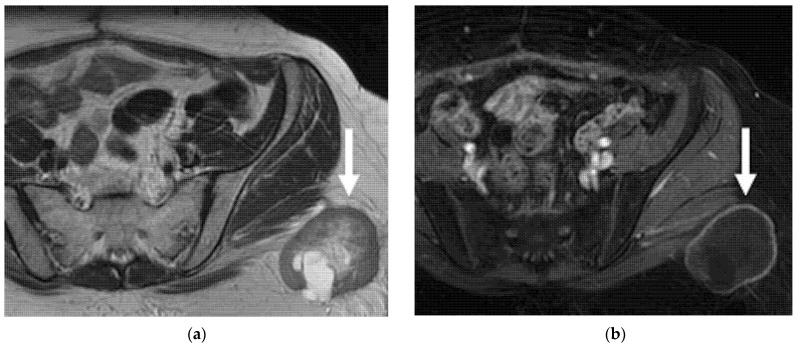
A 38-year-old woman who underwent radical hysterectomy with BSO and chemoradiation therapy for cervical cancer. (**a**) Axial FSE T2-weighted and (**b**) axial T1 weighted fat-sat post-contrast images show heterogeneous nodular lesion in the soft tissue of the left gluteal region (arrows). This finding was confirmed to be metastasis at histology.

In bone lesions, gadolinium-enhanced fat-suppressed T1WI may help the diagnosis by definition of foci of enhancement within the marrow space [29,37]. Other less common sites of recurrent disease have been described and include skin and subcutaneous tissues (Figure 11), brain, meninges, heart and breast. 

## 7. Conclusions

The major aim of surveillance is to improve survival and should be focused on early identification of recurrent disease to offer potentially curative treatment options to patients. In regard to cervical cancer, imaging surveillance may play a fundamental role in routine follow-up and usually includes MRI, CT and 18F-FDG PET/CT scans. MRI has been shown to be reliable in differentiating recurrence from post-treatment changes in previously treated cervical tumors. Furthermore, MRI is accurate in evaluation of recurrent tumor extent and organ invasion. Careful patient selection and a comprehensive imaging assessment of recurrent disease are of paramount importance when balancing the clinical benefits and risks derived from salvage therapies.

## Figures and Tables

**Table 1:** Imaging protocol.

CE-DW-MRI
T1-weighted spin-echo (SE) sequence
T2-weighted fast spin-echo (FSE) sequences in three imaging planes (axial/sagittal/coronal)
Fat saturated (FS) T2-weighted fast spin-echo (FSE) sequences in three imaging planes (axial/sagittal/coronal)
DWI in the axial plane using a single-shot spin-echo echo-planar imaging sequence with two b-values (0 and >500 s/mm^2^) with the same orientation and location used to acquire axial FSE T2-weighted images
Three-dimensional spoiled gradient-pulse FS T1-weighted before and after administration of 0.1 mmol/kg of gadolinium at a rate of 2 mL/s, which was followed by a 20-mL saline bolus injection

**Table 2:** Different types of recurrences.

Type of Recurrence	Imaging Findings
Local	
Cervix, parametria, or vaginal cuff 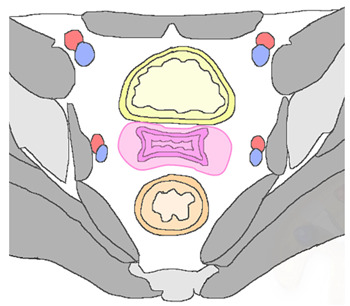	• On T2WI: o In the cervix: a region of intermediate-to-high SI, on a background of low SI change (radiation-induced fibrosis). o In the vagina (vaginal cuff or skip lesions): loss of the linear, low SI of the vaginal vault with an associated intermediate-to-high SI soft-tissue mass. • On post-contrast images: maximum tumor enhancement occurs between 45 and 90 s after contrast administration. • On DWI: an area with hyperintense signal on high-b-value images (with low ADC values on the ADC maps). • On contrast enhanced CT: a soft-tissue mass with early enhancement.
Regional	
Anterior Urinary bladder and urethra 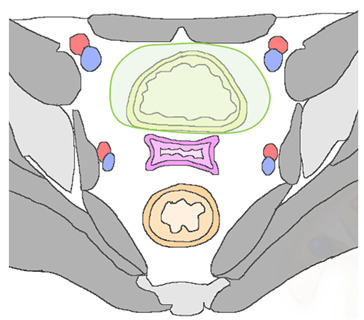	• On T2WI: o In the bladder: intermediate-to-high SI soft-tissue mass interrupting the normal low-signal-intensity of the bladder wall (probable muscle invasion), or the normal high SI of the mucosal layer (mucosal involvement). Vesicovaginal fistula appears as a linear fluid-filled tract, with disruption of the posterior bladder wall and infiltrative soft tissue into the bladder lumen or in peri-vesical space. o In the urethra: disruption of this bullseye aspect. • The use of post-contrast images and DWI may help differentiate bladder edema from true tumor infiltration. On post-contrast images fistulous track may be seen as linear peripheral enhancement connecting adjacent structures. • On excretory-phase CT scans contrast material may spread from the filling urinary bladder into the vaginal cuff or vagina. Central involvement of the bladder trigone at the ureteral orifice can cause ureteral obstruction, resulting in hydronephrosis.
Posterior Rectal or sigmoid colon 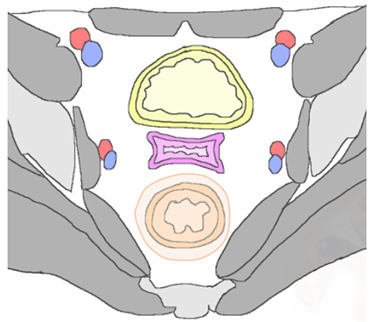	An infiltrating, spiculated mass, causing rectal or sigmoid luminal narrowing. On T2WI: disruption of the three layers characteristic of the intestinal wall by intermediate-to-high SI tumor, in absence of a clear fat plane. Fistulous tracks may demonstrate interruptions of the vaginal and rectum muscularis, with discontinuity of the intervening fat plane. Post-contrast images may help distinguish between post-radiotherapy inflammation and disease recurrence. In case of posterior pelvic recurrence, it is important to define whether the disease has reached and invaded the anal sphincter complex.
Lateral Pelvic sidewall 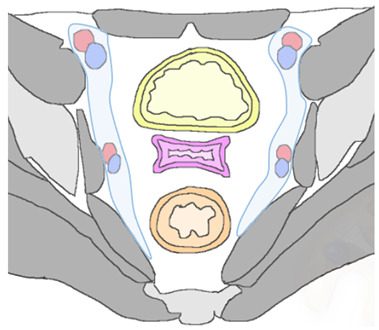	Pelvic sidewall recurrence is defined as a tumor extending within 3 mm and abutting the obturator internus or piriformis muscles with concomitant loss of fat planes. Other findings include: Iliac vessel invasion: vessels surrounded or distorted by tumor (degree of contact > 180°). Ureteral invasions: encased and narrowed ureter by tumor or ureteral obstruction at the level of the tumor. Sciatic nerve infiltration: obliteration of fat planes around the nerves on T1WI, and hyperintense SI at the nerve site on T2WI due to infiltrative edema. Enhancement of peri-neural soft tissue on post-contrast fat-suppressed T1WI is considered a sign of nerve involvement. Bony structures involvement: loss of low SI cortex on both T1 and T2WI, the replacement of the high SI T1W marrow by the intermediate-to-high SI tumor. On post-contrast images, a soft-tissue mass within the bone can be seen. On CT, the pelvic bones may show lytic destructive changes due to direct extension of tumor.
Lymphadenopathies	
Paracervical, parametrial, internal and external iliac, obturator, sacral, common iliac and para-aortic lymph nodes.	On MRI and post-contrast CT: enlarged lymph node with a short-axis diameter > 1 cm, a round shape, irregular margins, and internal heterogeneity. 18F-FDG PET/CT is better than the MRI or CT in detection of pathological lymph nodes.
Distant	
Supra-diaphragmatic lymph nodes	Supra-abdominal nodal recurrences include para-bronchial, supra-clavicular, axillary, mediastinal and hilar lymphadenopathy
Abdominal and extra-abdominal recurrence	Hepatic and adrenal involvement: image findings are non-specific and indistinguishable from involvement by other primary malignancies. Peritoneal carcinomatosis: implants scalloping the liver contour, peritoneal nodularity, and soft-tissue masses with extrinsic compression of bowel loops. Ascites often occurs. Lung: multiple pulmonary nodules, but they may also appear as a solitary nodule or present cavitation (squamous cell carcinoma). Osseous metastases: destruction of bone structure with a soft-tissue mass. Post-contrast images may help the diagnosis by definition of foci of enhancement within the marrow space.

MRI: magnetic resonance imaging; CT: computed tomography; T2WI or T1WI: T2 or T1 weighted imaging; DWI: diffusion-weighted imaging; SI: signal intensity.

## Data Availability

Not applicable.
